# Real‐world clinical practice of current periprocedural anticoagulation management in catheter ablation of atrial fibrillation: Data from a large prospective ablation registry

**DOI:** 10.1002/joa3.13182

**Published:** 2025-01-14

**Authors:** Yuta Taomoto, Shinsuke Miyazaki, Yasutoshi Nagata, Junichi Nitta, Osamu Inaba, Yasuhiro Shirai, Yasuaki Tanaka, Yukio Sekiguchi, Yukihiro Inamura, Yuichiro Sagawa, Akira Mizukami, Koji Azegami, Shinsuke Iwai, Hitoshi Hachiya, Yuichi Ono, Atsushi Takahashi, Takeshi Sasaki, Yasuteru Yamauchi, Hiroyuki Okada, Atsushi Suzuki, Makoto Suzuki, Keita Handa, Kenzo Hirao, Jun Nakajima, Takuro Nishimura, Susumu Tao, Masateru Takigawa, Tetsuo Sasano

**Affiliations:** ^1^ Department of Cardiology Japanese Red Cross Musashino Hospital Tokyo Japan; ^2^ Department of Cardiovascular Medicine Tokyo Medical and Dental University Tokyo Japan; ^3^ Department of Cardiology Sakakibara Heart Institute Tokyo Japan; ^4^ Department of Cardiology Japanese Red Cross Saitama Hospital Saitama Japan; ^5^ Department of Cardiology Disaster Medical Center Tokyo Japan; ^6^ Department of Cardiology Yokosuka Kyosai Hospital Kanagawa Japan; ^7^ Department of Cardiology Japanese Red Cross Yokohama City Bay Hospital Kanagawa Japan; ^8^ Department of Cardiology Kameda Medical Center Chiba Japan; ^9^ Department of Cardiology Shin‐Yurigaoka General Hospital Kanagawa Japan; ^10^ Department of Cardiology Hiratsuka Kyosai Hospital Kanagawa Japan; ^11^ Cardiovascular Center Tsuchiura Kyodo Hospital Ibaraki Japan; ^12^ Department of Cardiology Ome Municipal General Hospital Tokyo Japan; ^13^ Department of Cardiology Soka Municipal Hospital Saitama Japan; ^14^ Department of Cardiology Tokyo Yamate Medical Center Tokyo Japan; ^15^ Department of Cardiology Yokohama Minami Kyosai Hospital Yokohama Japan; ^16^ Division of Cardiology Kashiwa City Hospital Chiba Japan; ^17^ Arrhythmia Advanced Therapy Center AOI Universal Hospital Kanagawa Japan; ^18^ Department of Cardiology Tokyo Metropolitan Toshima Hospital Tokyo Japan

**Keywords:** anticoagulation, atrial fibrillation, catheter ablation

## Abstract

**Background:**

The guidelines recommend anticoagulation management with uninterrupted warfarin or direct thrombin inhibitors (DTIs) during the atrial fibrillation (AF) ablation periprocedural period.

**Objectives:**

To clarify the Japanese real‐world latest periprocedural anticoagulation management during AF ablation.

**Methods:**

This multicenter observational study included 6232 consecutive AF patients (68.7 ± 10.9 years, 4346 men) who underwent periprocedural anticoagulation therapy using direct oral anticoagulants (DOACs) between January 2022 and August 2023.

**Results:**

The mean CHADS_2_ and CHA_2_DS_2_VASc scores were 1.2 ± 1.1 and 2.3 ± 1.5. Bleeding and thromboembolic events occurred in 79 (1.3%) and eight (0.12%) patients. During the periprocedural period, factor Xa inhibitors (FXaIs) were used in 3063 patients (rivaroxaban in 624, apixaban in 1093, and edoxaban in 1345) and DTIs in 3170 including 2583 in whom DTIs were switched from FXaIs. Both the bleeding (0.85% vs. 1.69%, *p* = .003) and thromboembolic event rates (0.03% vs. 0.23%, *p* = .036) were significantly lower in the DTI‐ than FXaI‐group. A multivariate analysis showed periprocedural FXaI use was significantly associated with both bleeding events (odds ratio [OR] = 1.92, 95% confidence interval [CI] = 1.20–3.08, *p* = .006) and cardiac tamponade (OR = 2.74, 95% CI = 1.27–5.9, *p* = .01). The interval between the last DOAC administration and the procedure was significantly shorter in the DTI‐ than FXaI‐group (4.2 ± 4.9 vs. 19.3 ± 10.7 h, *p* < .01). In the FXaI‐group, the bleeding rate tended to be lower in the minimally interrupted (*n* = 2105) than uninterrupted group (*n* = 821) (1.47% vs. 2.56%, *p* = .06). Two patients in the uninterrupted FXaI‐group required surgical management for cardiac tamponade.

**Conclusions:**

Our multicenter real‐world data demonstrated that anticoagulation with DTIs was a reasonable periprocedural anticoagulation regimen to reduce periprocedural complications.

## Graphical abstract

Anticoagulation with direct thrombin inhibitors was a reasonable periprocedural anticoagulation regimen to reduce periprocedural complications in atrial fibrillation ablation.

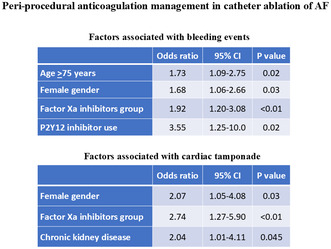

## 1 INTRODUCTION

Atrial fibrillation (AF) is the most common arrhythmia and anticoagulation therapy is recommended based on the stroke risk assessment. After the introduction of direct oral anticoagulants (DOACs), most AF patients have undergone anticoagulation therapy with DOACs instead of warfarin because of the favorable safety and efficacy, and better pharmacokinetic profile, including short half‐lives, ease of administration, fewer interactions, and no need for laboratory monitoring. Catheter ablation has become an established treatment of AF, and its indication has greatly expanded owing to the favorable clinical outcomes. However, periprocedural thromboembolic and hemorrhagic events remain the most worrisome complications, and periprocedural anticoagulation management plays a key role in anticipating them.^1^, ^2^ Previous randomized clinical trials have demonstrated that (1) uninterrupted warfarin significantly reduces thromboembolic events more than interrupted warfarin with a heparin bridge,^3^ (2) thromboembolic and hemorrhagic events are similar with uninterrupted factor Xa inhibitors (FXaIs) and uninterrupted warfarin,^4^, ^5^, ^6^ and (3) the uninterrupted direct thrombin inhibitor (DTI), dabigatran, significantly reduces bleeding events more than uninterrupted warfarin with equivalent thromboembolic events.^7^ According to that evidence, the latest guidelines recommend uninterrupted warfarin or DTIs as class 1 (evidence level A), uninterrupted Xa inhibitors as class 2a, and minimally interrupted DOACs as class 2a, as a periprocedural anticoagulation management of AF ablation.^2^ However, in the real‐world, most patients are anticoagulated with FXaIs, but not warfarin or DTIs.^8^ As a result, to follow the class 1 guideline recommendation, switching from FXaIs to a DTI is required before the procedure. In this study, we sought to clarify the current real‐world periprocedural anticoagulation management during AF ablation in Japan by analyzing the latest multicenter prospective ablation registry data.

## 2 METHODS

### 2.1 Study design and population

This was an observational cohort study. A total of 6503 consecutive AF ablation procedures in 20 Japanese cardiovascular centers between January 2022 and August 2023 were included. Patients were grouped according to the type of anticoagulants received periprocedurally. The data were extracted from the TMDU (Tokyo Medical and Dental University)‐ablation registry (Clinical trial registry, UMIN 000047063), in which all cases who underwent catheter ablation at 20 Japanese centers had been prospectively registered from January 2022, and the data of the patient characteristics, procedural details, and clinical outcomes were collected via an electronic data capture system.^9^ The consent for the procedure was obtained by written informed consent. The need for patient consent was waived because of the anonymized nature of the study (through an opt‐out procedure). The study was approved by each hospital's institutional review board. AF was classified according to the latest guidelines.^1^, ^2^ The study complied with the Declaration of Helsinki.

### 2.2 Ablation protocol

Pre‐procedural transesophageal echocardiography or contrast cardiac computed tomography was performed prior to the procedure to exclude left atrial thrombi. The procedure was performed under moderate or deep sedation. Anticoagulation management was according to the latest guidelines and an activated clotting time of ≥300 was maintained throughout the procedure. Following a transseptal puncture, pulmonary vein isolation (PVI) was performed with balloon systems (mainly the cryoballoon), or radiofrequency catheters under the guidance of three‐dimensional mapping systems. During the initial procedure, the procedural endpoint was defined as an electrical PVI. Additional ablation beyond the PVI was performed according to the operators' discretion. During repeat procedures, a re‐PVI was consolidated initially, then additional ablation was performed.

### 2.3 Study outcomes

The primary outcome was defined as periprocedural bleeding events during hospitalization. Bleeding events were adjudicated as defined by the Bleeding Academic Research Consortium (BARC).^10^ Major bleeding was defined as bleeding events with a BARC2‐5. The secondary outcome was defined as thromboembolic events during hospitalization. It was diagnosed with magnetic resonance imaging and/or computed tomography by a neurologist in each institute.

The anticoagulants were classified as warfarin, FXaIs (apixaban, edoxaban, and rivaroxaban), and DTIs (dabigatran). Since DTIs are recommended as a class 1 indication in the guidelines,^2^ FXaIs were switched to a DTI before the procedure (at least 1 day before the procedure) at the operator's discretion. FXaIs might be minimally interrupted during the periprocedural period at the operator's discretion (class 2a indication). The patients with an additional heparin bridge during the periprocedural period were excluded from the analysis. The anticoagulation therapy after the procedure was decided at the operator's discretion, however in general, the same anticoagulants (including switched anticoagulants) were continued for at least 1 month after the procedure.

### 2.4 Statistical analysis

Continuous data are expressed as the mean ± standard deviation for normally distributed variables or as the median [25th, 75th percentiles] for non‐normally distributed variables, and were compared using a student's *t*‐test or Mann–Whitney *U*‐test, respectively. Categorical variables were compared using the Chi‐squared test or Fisher's exact test when the number of events was less than 5. Statistical significance was set at *p* < .05. A multivariate logistic regression analysis was performed to identify the factors associated with bleeding events and cardiac tamponade. The final predictive model was selected using the backward stepwise variable selection procedure based on the Akaike information criterion. All statistical analyses were performed with EZR software (Saitama Medical Center, Jichi Medical University, Saitama, Japan), which is a graphical user interface for R (The R Foundation for Statistical Computing, Vienna, Austria). More precisely, it is a modified version of R commander designed to add statistical functions frequently used in biostatistics.^11^

## 3 RESULTS

### 3.1 Study population and bleeding/thromboembolic events

Among 6503 consecutive AF ablation procedures, warfarin was used during the periprocedural period in 206 (3.2%) and the data were missing in 65 (0.99%). The remaining 6232 (96.8%) patients who underwent AF ablation with DOACs during the periprocedural period were analyzed. The mean age was 68.7 ± 10.9 years, 4346 (69.7%) patients were men, and the creatinine clearance was 73.9 ± 27.3 mL/min. Three‐hundred seventy‐six (6.0%) patients had a history of a cerebral infarction, and the mean CHADS_2_ and CHA_2_DS_2_VASc scores were 1.2 ± 1.1 and 2.3 ± 1.5, respectively. The baseline anticoagulants were dabigatran, rivaroxaban, apixaban, and edoxaban in 770 (12.4%), 1049 (16.8%), 1820 (29.2%), and 2593 (41.6%) patients, respectively.

Major bleeding events occurred in a total of 79 (1.3%) patients, a BARC 2 in 38 patients (16 in the DTI group and 22 in the FXaI group), a BARC 3a in five (one in the DTI group and four in the FXaI group), and a BARC 3b in 36 (10 in the DTI group and 26 in the FXaI group) including cardiac tamponade in 34 (nine in the DTI group and 25 in the FXaI group). Thromboembolic events occurred in eight (0.12%) patients, including cerebral infarctions in seven.

### 3.2 Direct thrombin inhibitors versus factor Xa inhibitors

FXaIs were switched to a DTI before the procedure in 2583 (41.4%) patients. During the periprocedural period, DTIs were used in 3170 (50.9%) patients, while FXaIs were used in the remaining 3062 (49.1%). The patients in the DTI group were younger, more male‐dominated, and had lower CHADS_2_ and CHA_2_DS_2_VASc scores than those in the FXaIs group (Table 1). The procedural parameters are presented in Table S1.

**TABLE 1:** Patient characteristics in the thrombin inhibitor and factor Xa inhibitors groups.

	Thrombin inhibitor	Factor Xa inhibitors	*p* value
	*n* = 3170	*n* = 3062	
Age, years	68.1 ± 11.0	69.2 ± 10.8	<.01
Male gender, *n* (%)	2281 (72.0%)	2065 (67.4%)	<.01
Body mass index, kg/m^2^	24.4 ± 3.9	24.1 ± 3.9	<.01
Creatinine clearance, mL/min	76.1 ± 27.1	71.7 ± 27.5	<.01
Hemoglobin, g/dL	14.1 ± 1.6	14.0 ± 1.6	.12
Hypertension, *n* (%)	1492 (47.1%)	1589 (51.9%)	<.01
Diabetes, *n* (%)	409 (12.9%)	488 (15.9%)	<.01
Heart failure, *n* (%)	506 (16.0%)	591 (19.3%)	<.01
Cerebral infarction, *n* (%)	194 (6.1%)	182 (5.9%)	.79
CHADS_2_ score	1.2 ± 1.1	1.3 ± 1.1	<.01
CHA_2_DS_2_‐VASc score	2.1 ± 1.5	2.4 ± 1.5	<.01
Left atrial diameter, mm	40.2 ± 7.0	39.7 ± 6.9	<.01
Left ventricular ejection fraction, %	62.9 ± 11.0	60.4 ± 10.8	.50
Aspirin use, *n* (%)	52 (1.6%)	63 (2.1%)	.26
P2Y12 inhibitor use, *n* (%)	51 (1.6%)	47 (1.5%)	.84
Baseline DOACs			
Dabigatran, *n* (%)	770 (24.3%)	0 (0%)	
300 mg, *n* (%)	392 (12.4%)	0 (0%)	
220 mg, n (%)	378 (11.9%)	0 (0%)	
Apixaban, *n* (%)	727 (22.9%)	1093 (35.7%)	
10 mg, *n* (%)	626 (19.7%)	922 (30.1%)	
5 mg, *n* (%)	101 (3.2%)	171 (5.6%)	
Edoxaban, *n* (%)	1248 (39.3%)	1345 (46.4%)	
60 mg, *n* (%)	64 (2.0%)	20 (0.7%)	
30 mg, *n* (%)	498 (15.7%)	686 (22.4%)	
15 mg, *n* (%)	686 (21.6%)	639 (20.9%)	
Rivaroxaban, *n* (%)	425 (13.4%)	624 (20.4%)	
15 mg, *n* (%)	56 (1.8%)	94 (3.1%)	
10 mg, *n* (%)	369 (11.6%)	530 (17.3%)	

Abbreviations: DOAC, direct oral anticoagulant; *n*, number.

The prevalence of bleeding events (0.85% vs. 1.69%, *p* = .003) and cardiac tamponade (0.28% vs. 0.82%, *p* = .005) were significantly lower in the DTI group than FXaI group. In the multivariate logistic regression analysis, being in the FXaI group (odds radio [OR] = 1.92, 95% confidence interval [CI] =1.20–3.08, *p* < .01), age ≥75 years (OR = 1.73, 95% CI = 1.09–2.75, *p* = .02), female gender (OR = 1.68, 95% CI = 1.06–2.66, *p* = .03), and P2Y12 inhibitor use (OR = 3.55, 95% CI = 1.25–10.0, *p* = .02) were significant predictors of bleeding events (Table 2A). Similarly, being in the FXaI group (OR = 2.74, 95% CI = 1.27–5.9, *p* < .01), female gender (OR = 2.07, 95% CI = 1.05–4.08, *p* = .03), and chronic kidney disease (estimated glomerular filtration rate < 60 mL/min/1.73m^2^) (OR = 2.04, 95% CI = 1.01–4.11, *p* = .045) were significant predictors of cardiac tamponade (Table 2(B)). The results of the univariate analysis are presented in Table S2. The incidence of thromboembolic events was also significantly lower in the DTI group than FXaI group (0.03% vs. 0.23%, *p* = .036).

**TABLE 2:** Statistically significant factors associated with bleeding events (A) and cardiac tamponade (B) in multivariate analysis.

	Odds ratio	95% CI	*p* value
A.			
Age ≥75 years	1.73	1.09–2.75	.02
Female (gender)	1.68	1.06–2.66	.03
Factor Xa inhibitors group	1.92	1.20–3.08	<.01
P2Y12 inhibitor use	3.55	1.25–10.0	.02
B.			
Female gender	2.07	1.05–4.08	.03
Factor Xa inhibitors group	2.74	1.27–5.90	<.01
Chronic kidney disease	2.04	1.01–4.11	.045

Abbreviations: CI, confidence interval.

Among the 34 patients who required pericardiocentesis for cardiac tamponade, surgical intervention was required in two patients in the FXaI group but in no patients in the DTI group (8.0% vs. 0%, *p* = 1.0). Blood transfusions were required in four patients in the FXaI group but in no patients in the DTI group (16.0% vs. 0%, *p* = .55). Though the FXaI reversal agent was approved in May 2022, it was not used in any patients. In the DTI group, idarucizumab was administered in five (55.5%) patients. There was no significant difference in the volume of drainage between the DTI group and FXaI group (254 ± 154 vs. 321 ± 190 mL, *p* = .36).

### 3.3 Factor Xa inhibitors and events

Among the 3062 patients in the FXaI group, rivaroxaban, apixaban, and edoxaban were used in 624 (20.4%), 1093 (35.7%), and 1345 (46.4%) patients, respectively (Table 3). The rivaroxaban group was younger, more male‐dominated, and had a higher creatinine clearance. There was no significant difference in the prevalence of bleeding events (1.9% in the rivaroxaban, 1.6% in the apixaban, and 1.6% in the edoxaban group, *p* = .89). The incidence of thromboembolic events was similar among the three groups (0.2% in the rivaroxaban, 0.1% in the apixaban, and 0.3% in the edoxaban group, *p* = .89).

**TABLE 3:** Patient characteristics in the factor Xa group.

	Apixaban	Edoxaban	Rivaroxaban	*p* value
	*n* = 1093	*n* = 1345	*n* = 624	
Age, years	69.5 ± 10.6	69.7 ± 10.8	67.6 ± 10.9	<.01
Male gender, *n* (%)	759 (69.4%)	825 (61.3%)	481 (77.1%)	<.01
Body mass index, kg/m^2^	24.2 ± 4.0	23.6 ± 3.9	24.8 ± 3.9	<.01
Creatinine clearance, mL/min	70.3 ± 27.2	70.1 ± 27.7	77.7 ± 26.6	<.01
Hemoglobin, g/dL	14.0 ± 1.6	14.0 ± 4.6	14.3 ± 1.5	.07
Hypertension, *n* (%)	563 (51.5%)	670 (49.8%)	356 (57.1%)	.01
Diabetes, *n* (%)	182 (16.7%)	197 (14.6%)	109 (17.5%)	.20
Heart failure, *n* (%)	235 (21.5%)	245 (18.2%)	111 (17.8%)	.07
Cerebral infarction, *n* (%)	76 (7.0%)	71 (5.3%)	35 (5.6%)	.20
CHADS_2_ score	1.4 ± 1.1	1.3 ± 1.1	1.3 ± 1.1	.19
CHA_2_DS_2_‐VASc score	2.4 ± 1.5	2.4 ± 1.5	2.2 ± 1.5	<.01
Left atrial diameter, mm	40.4 ± 6.7	38.9 ± 6.8	40.0 ± 7.2	<.01
Left ventricular ejection fraction, %	59.2 ± 11.9	61.1 ± 10.4	61.1 ± 9.6	<.01
Aspirin use, *n* (%)	20 (1.8%)	29 (2.2%)	14 (2.2%)	.80
P2Y12 inhibitor use, *n* (%)	20 (1.8%)	20 (1.5%)	7 (1.1%)	.51

Abbreviation: *n*, number.

### 3.4 Uninterrupted versus minimally interrupted Xa inhibitors

During the periprocedural period, FXaIs were uninterrupted in 821 (28.1%) patients, while they were minimally interrupted (skip once in the morning) in the remaining 2105 (71.9%). The 136 patients in whom a heparin bridge was performed during the periprocedural period were excluded from the analysis. The patients in the minimally interrupted group were significantly younger, had lower CHADS_2_ and CHA_2_DS_2_VASc scores, and had a smaller left atrial size (Table 4).

**TABLE 4:** Patient characteristics in the minimally interrupted and uninterrupted factor Xa groups.

	Minimally interrupted	Uninterrupted	*p* value
	*n* = 2105	*n* = 821	
Age, years	68.8 ± 10.8	70.0 ± 10.7	<.01
Male gender, *n* (%)	1431 (68.0%)	541 (65.9%)	.29
Body mass index, kg/m^2^	24.1 ± 3.9	24.1 ± 3.9	.83
Creatinine clearance, mL/min	72.6 ± 27.2	69.8 ± 27.9	.014
Hemoglobin, g/dL	14.1 ± 1.6	13.9 ± 1.6	<.01
Hypertension, *n* (%)	1068 (50.7%)	43 (54.0%)	.12
Diabetes, *n* (%)	328 (15.6%)	138 (16.8%)	.43
Heart failure, *n* (%)	372 (17.7%)	191 (23.3%)	<.01
Cerebral infarction, *n* (%)	117 (5.6%)	55 (6.7%)	.26
CHADS_2_ score	1.3 ± 1.1	1.5 ± 1.1	<.01
CHA_2_DS_2_‐VASc score	2.3 ± 1.5	2.5 ± 1.5	<.01
Left atrial diameter, mm	39.4 ± 7.1	40.2 ± 6.2	<.01
Left ventricular ejection fraction, %	59.6 ± 10.6	62.3 ± 11.3	<.01
Aspirin use, *n* (%)	50 (2.4%)	11 (1.3%)	.08
P2Y12 inhibitor use, *n* (%)	33 (1.6%)	13 (1.6%)	1.00
DOACs type			.118
Apixaban, *n* (%)	756 (35.9%)	276 (33.6%)	
Edoxaban, *n* (%)	917 (43.6%)	378 (46.0%)	
Rivaroxaban, *n* (%)	432 (20.5%)	167 (20.3%)	

Abbeviations: *n*, number.

The prevalence of bleeding events tended to be lower in the minimally interrupted group than uninterrupted group, but the difference did not reach a statistical significance (1.47% vs. 2.56%, *p* = .06). Surgical management was required for cardiac tamponade in the uninterrupted apixaban and edoxaban groups in one patient each. There was no statistically significant difference in the prevalence of thromboembolic events between the uninterrupted and minimally interrupted groups (0.12% vs. 0.28%, *p* = .68).

### 3.5 The interval between the last DOAC administration and the procedure

In the DTI group, DTIs were uninterrupted in the vast majority of the patients (3050 [96.2%]). The interval between the last DOAC administration and the procedure (D‐P interval) was significantly shorter in the DTI group than FXaI group (4.2 ± 4.9 vs. 19.3 ± 10.7, *p* < .01). The D‐P interval was similar between the patients with bleeding events and those without in the DTI group (4.2 ± 4.9 vs. 4.5 ± 4.9 h, *p* = .75), but was significantly shorter in the patients with bleeding events than in those without in the FXaI group (16.3 ± 10.4 vs. 19.3 ± 10.7 h, *p* = .04). In the FXaI group, there was no significant difference in the D‐P interval between the patients with thromboembolic events and those without (20.3 ± 8.6 vs. 19.3 ± 10.7 h, *p* = .80). In the DTI group, the D‐P interval was 1 h in one patient with a thromboembolic event.

## 4 DISCUSSION

Our real‐world data of periprocedural anticoagulation therapy found that (1) FXaI use was significantly associated with major bleeding events and cardiac tamponade, (2) the thromboembolic event rate was higher in the FXaI than DTI group, and the D‐P interval was significantly longer in the FXaI than DTI group, (3) in the FXaI group, the D‐P interval was significantly shorter in the patients with bleeding events than in those without, (4) idarucizumab was used in the majority of the patients with cardiac tamponade in the DTI group, and (5) surgical intervention was required for cardiac tamponade only in the uninterrupted FXaI groups without an FXaI reversal agent.

### 4.1 Real‐world data of periprocedural anticoagulation management

A bleeding complication is an important concern with anticoagulation therapy given the close relationship with morbidity and mortality. Currently, DOACs are recommended in terms of warfarin, especially in the Asian population with a relatively high risk of bleeding with warfarin.^12^, ^13^, ^14^ In terms of the periprocedural anticoagulation during AF ablation, a meta‐analysis including 6827 Japanese patients from 19 studies between 2012 and 2019 showed that DOACs resulted in a lower risk of bleeding complications than warfarin.^15^ Another retrospective cross‐sectional study that included 6564 patients from a nationwide Japanese administrative claims database between 2011 and 2020 demonstrated significantly lower thromboembolic events in the DOAC than warfarin group.^16^ Because of the several advantages of DOACs over warfarin, as shown in this study, most periprocedural anticoagulants are DOACs at present. Indeed, in a multicenter observational study including 3072 Japanese patients between 2017 and 2018, 92.6% of the patients took DOACs, however, 82.3% of them still received minimally interrupted DOACs periprocedurally despite being a class 2a recommendation.^8^ In the present study, 72% of the FXaI group patients similarly minimally skipped anticoagulants. It is likely that physicians minimally skip DOACs out of concern that bleeding complications could lead to worse outcomes under a lack of reversal agents.

### 4.2 Direct thrombin inhibitors as a periprocedural anticoagulant

DOACs are categorized as DTIs or FXaIs based on their targets. A recommended anticoagulant as a class 1 indication other than warfarin is a DTI alone.^2^ That is because (1) only uninterrupted DTIs significantly reduce the periprocedural major bleeding events as compared to warfarin,^7^ and (2) a specific reversal agent (idarucizumab), which is a humanized monoclonal antibody fragment that binds to DTIs, can completely reverse the anticoagulant effect of DTIs within minutes.^17^ The mechanism of reducing major bleeding events with DTIs may be related to the more specific mechanism of action (thrombin inhibition rather than factor Xa inhibition). Our real‐world data demonstrated that periprocedural FXaI use was significantly associated with major bleeding events and cardiac tamponade in clinical practice. Switching to a DTI from an FXaI should make managing severe bleeding complications easier for the physicians and would seem to be a reasonable strategy. Indeed, the severity of cardiac tamponade was lower in the DTI group, and surgical management was required only in the FXaI group. Nevertheless, still half of the procedures were performed under anticoagulation therapy with FXaIs in the real‐world setting. We speculated the reasons were as follows: (1) changing anticoagulants from FXaIs to DTIs is simply troublesome for physicians in daily practice, (2) DTIs are capsules that are difficult to swallow in a part of the population and may cause dyspepsia, and (3) DTIs are difficult to use in patients with moderate‐to‐severe renal dysfunction.

Regarding the thromboembolic complications, because of the low event rate, most studies including randomized control trials were underpowered for making definitive assumptions on the efficacy of an anticoagulation strategy over other strategies. In the present study, the thromboembolic event rate was higher in the FXaI group than the DTI group. This might be explained by the fact that the D‐P interval was significantly longer in the FXaI group than the DTI group.

### 4.3 Factor Xa inhibitors

The feasibility of a minimally interrupted DOAC therapy, which is a class 2a indication, is still under debate. Meta‐analyses have demonstrated no significant difference in bleeding and thromboembolic outcomes with an uninterrupted versus interrupted DOAC therapy except for a lower risk of silent strokes in the uninterrupted DOAC therapy.^18^, ^19^, ^20^ In clinical practice, FXaIs are still minimally interrupted in the majority of the population owing to the concern for periprocedural bleeding events. Actually, in the FXaI group, the D‐P interval was significantly shorter in the patients with bleeding events than in those without. Moreover, two patients in the uninterrupted FXaI group experienced a cardiac tamponade requiring surgical management. That data supported why the physicians preferred a minimally interrupted DOAC therapy in the FXaI group.

In May 2022, an FXaI reversal agent, andexanet alfa, which acts as a decoy and inhibits FXaIs from binding to natural factor Xa, became available in Japan. However, the higher frequency of thromboembolic events than idarucizumab presumably because of the increasing thrombin generation, high cost of the medicine, and complexity of the dosing regimen are major concerns.^21^ Therefore, andexanet alfa still has not become widely available in Japanese hospitals.

The strength of the present data included the sufficiently large population to assess the low incidence of complications, the latest data sets in the era of DOACs (from 2022 to 2023), multicenter data (20 ablation centers), and real‐world data including all consecutive patients undergoing AF ablation (low selection bias). According to the conclusions of this paper, we preferred DTIs with a relatively high efficacy and fewer bleeding complications than FXaIs as the periprocedural anticoagulant regimen during AF ablation.

### 4.4 Study limitations

As this study captured the real‐world data, there was no rigorous anticoagulant protocol, and the choice of the anticoagulation regimen was left to the operators' discretion. Owing to the latest dataset, only the acute procedural data were available. The cause of the cardiac tamponade was multifactorial and was undetermined in this study.

## 5 CONCLUSIONS

Our multicenter real‐world data demonstrated that anticoagulation using DTIs was the most reasonable periprocedural anticoagulation regimen during AF ablation to minimize the incidence of bleeding and thromboembolic events.

## FUNDING INFORMATION

None.

## CONFLICT OF INTEREST STATEMENT

The authors declare no conflicts of interest.

## DISCLOSURES

Dr. Miyazaki received speaker honoraria from Bristol‐Meyers Squibb, Daiichi Sankyo, and Boehringer Ingelheim.

## ETHICS STATEMENT

The study was approved by each hospital's institutional review board.

## CONSENT

Informed consent for this study was obtained through an opt‐out form in accordance with the Declaration of Helsinki.

## Supporting information


Data S1.

Data S2.

## Data Availability

The datasets used in the current study are available from the corresponding author upon reasonable request.
