# Supplementary material for: Real‐world clinical practice of current periprocedural anticoagulation management in catheter ablation of atrial fibrillation: Data from a large prospective ablation registry
Source: J Arrhythm. 2025 Jan 14;41(1):e13182. doi: 10.1002/joa3.13182 (PMC11730986; doi:10.1002/joa3.13182)
Supplement: Supplementary file 1 — Data S1. [file JOA3-41-e13182-s002.docx]

**Supplementary Table 1. Procedural parameters**

|  | Thrombin inhibitor | Factor Xa inhibitors | *P* value |
| --- | --- | --- | --- |
| Total procedure time, mins | 148 ± 72 | 131 ± 61 | <0.001 |
| PVI, n (%) | 2438 (76.9%) | 2427(79.4%) | 0.03 |
| Cryoballoon use, n (%) | 875 (36.6%) | 1781 (73.1%) | <0.001 |
| re-PVI, n (%) | 376 (11.9%) | 286 (9.4%) | 0.001 |
| SVC isolation, n (%) | 541 (17.1%) | 633 (20.7%) | <0.001 |
| CTI ablation, n (%) | 1870 (59.0%) | 1469 (48.0%) | <0.001 |
| non-PV foci, n (%) | 202 (6.4%) | 180 (5.9%) | 0.43 |
| LA roof line, n (%) | 1256 (39.6%) | 1150 (37.6%) | 0.10 |
| Mitral isthmus line, n (%) | 220 (6.9%) | 385 (12.6%) | <0.001 |
| Bottom line, n (%) | 1076 (33.9%) | 525 (17.1%) | <0.001 |
| Anterior wall line, n (%) | 54 (1.7%) | 49 (1.6%) | 0.77 |
| VOM chemical ablation, n (%) | 114 (3.6%) | 287 (9.4%) | <0.001 |
| FAAM ablation, n (%) | 21 (0.7%) | 76 (2.5%) | <0.001 |
| CFAE ablation, n (%) | 13 (0.4%) | 57 (1.9%) | <0.001 |

CFAE, complex fractionated atrial electrograms; CTI, cavo-tricuspid isthmus; FAAM, Fractionated signal area of atrial muscle; LA, left atrial; PV(I), pulmonary vein (isolation); SVC, superior vena cava; VOM, vein of Marshall.
