# Supplementary material for: Real‐world clinical practice of current periprocedural anticoagulation management in catheter ablation of atrial fibrillation: Data from a large prospective ablation registry
Source: J Arrhythm. 2025 Jan 14;41(1):e13182. doi: 10.1002/joa3.13182 (PMC11730986; doi:10.1002/joa3.13182)
Supplement: Supplementary file 2 — Data S2. [file JOA3-41-e13182-s001.docx]

**Supplementary Table 2. Factors associated with bleeding events (A) and cardiac tamponade (B) in univariate analysis**

**A.**

|  | Hazard ratio | 95% CI | *P* value |
| --- | --- | --- | --- |
| P2Y12 inhibitor use | 3.75 | 1.3-10.8 | 0.01 |
| Factor Xa inhibitor group | 1.86 | 1.16-2.99 | 0.009 |
| Age >75 years | 1.55 | 0.96-2.52 | 0.07 |
| Female gender | 1.57 | 0.98-2.52 | 0.05 |
| Chronic kidney disease | 1.4 | 0.87-2.22 | 0.17 |
| Hypertension | 1.4 | 0.88-2.25 | 0.16 |
| OCI/TIA | 1.34 | 0.48-3.73 | 0.57 |
| Diabetes mellitus | 0.99 | 0.51-1.92 | 0.98 |
| Body mass index | 0.962 | 0.90-1.02 | 0.23 |
| Aspirin use | 0.531 | 0.07-3.97 | 0.54 |

**B.**

|  | Hazard ratio | 95% CI | *P* value |
| --- | --- | --- | --- |
| P2Y12 inhibitor use | <0.0001 | - | 0.99 |
| Factor Xa inhibitor group | 2.76 | 1.28-5.94 | 0.0097 |
| Age >75 years | 1.69 | 0.81-3.5 | 0.16 |
| Female gender | 1.93 | 0.96-3.89 | 0.06 |
| Chronic kidney disease | 1.82 | 0.88-3.75 | 0.11 |
| Hypertension | 1.87 | 0.91-3.84 | 0.09 |
| OCI/TIA | <0.0001 | - | 0.99 |
| Diabetes mellitus | 0.909 | 0.34-2.42 | 0.85 |
| Body mass index | 1.04 | 0.95-1.13 | 0.44 |
| Aspirin use | <0.0001 | - | 0.99 |

CI, confidence interval; OCI, old cerebral infarction; TIA, transient ischemic attack.
